# Monitoring and evaluation of anesthesia depth status data based on neuroscience

**DOI:** 10.1515/biol-2022-0719

**Published:** 2023-11-21

**Authors:** Yuhua Bi, Junping Huang, Mei Li, Siying Li, Heshou Lei

**Affiliations:** Department of Anesthesiology, Wuming Hospital Affiliated to Guangxi Medical University, Nanning 530199, Guangxi, China

**Keywords:** depth of anesthesia, neuroscience technology, anesthesia status monitoring algorithm, brain neural activity, drug concentration, degree of blood oxygen saturation

## Abstract

Monitoring and analysis of anesthesia depth status data refers to evaluating the anesthesia depth status of patients during the surgical process by monitoring their physiological index data, and conducting analysis and judgment. The depth of anesthesia is crucial for the safety and success of the surgical process. By monitoring the state of anesthesia depth, abnormal conditions of patients can be detected in a timely manner and corresponding measures can be taken to prevent accidents from occurring. Traditional anesthesia monitoring methods currently include computer tomography, electrocardiogram, respiratory monitoring, etc. In this regard, traditional physiological indicator monitoring methods have certain limitations and cannot directly reflect the patient’s neural activity status. The monitoring and analysis methods based on neuroscience can obtain more information from the level of brain neural activity. Purpose: In this article, the monitoring and analysis of anesthesia depth status data would be studied through neuroscience. Methods: Through a controlled experiment, the monitoring accuracy of traditional anesthesia status monitoring algorithm and neuroscience-based anesthesia status monitoring algorithm was studied, and the information entropy and oxygen saturation of electroencephalogram signals in patients with different anesthesia depth were explored. Results: The experiment proved that the average monitoring accuracy of the traditional anesthesia state monitoring algorithm in patients’ blood drug concentration and oxygen saturation reached 95.55 and 95.00%, respectively. In contrast, the anesthesia state monitoring algorithm based on neuroscience performs better, with the average monitoring accuracy of drug concentration and oxygen saturation reaching 98.00 and 97.09%, respectively. This experimental result fully proved that the monitoring performance of anesthesia state monitoring algorithms based on neuroscience is better. Conclusion: The experiment proved the powerful monitoring ability of the anesthesia state monitoring algorithm based on neuroscience used in this article, and explained the changing trend of brain nerve signals and oxygen saturation of patients with different anesthesia depth states, which provided a new research method for the monitoring and analysis technology of anesthesia depth state data.

## 1 Introduction

Anesthesia is a medical practice that involves administering medication to patients during surgical procedures to keep them unconscious or reduce their sensitivity to pain. Anesthesia plays a vital role in modern medicine, allowing surgical procedures to be performed safely and effectively. Otherwise, these surgeries can be very painful or dangerous. To ensure the safety and effectiveness of anesthesia, it is important to monitor the patient’s anesthesia depth throughout the entire surgical process. This process helps anesthesiologists accurately evaluate the level of sedation, analgesia, and amnesia required to maintain the appropriate level of anesthesia during the surgical process. By closely monitoring and adjusting the depth of anesthesia for patients, anesthesiologists can minimize the risk of side effects and complications related to anesthesia, and promote faster and more successful postoperative recovery. As an emerging technology, neuroscience has a wide range of applications and potential. Here are some of the main applications: brain science, neural engineering, psychology, psychiatry, etc. It can provide better healthcare, scientific research, and human welfare services for people.

Based on the relevant information consulted, the following scholars’ research on anesthesia technology is listed. Pandya et al. studied the origin, evolution, and dissemination of anesthesia monitoring standards. He documented the significant changes in medical practice caused by the activities of a small group of anesthesiologists at Harvard Affiliated Hospital, ending with the current strategy of minimizing anesthesia-related risks. In addition, he also consulted public domain documents and major source materials, which led to the establishment of Harvard University’s groundbreaking minimum monitoring anesthesia monitoring standards [1]. Shander pointed out that anesthesia practice has historically been rooted in the management needs for issues such as pain and consciousness, which belong to the field of the nervous system, and many anesthetics play a major role in the brain. In addition, he discussed the challenges of monitoring brain function during anesthesia. Although brain function is an important aspect of anesthesia, technological limitations make monitoring brain function more difficult. In contrast, the functions of the heart, circulatory system, blood, lungs, and kidneys are easier to understand and measure [2]. Romagnoli believed that individual responses to sedatives and hypnotic are highly variable, and determining personalized doses during anesthesia in the operating room and sedation in the intensive care unit may have beneficial effects. He discussed the importance of electroencephalography in exploring anesthesia and sedative responses. The processed electroencephalogram (EEG) provides easy-to-use indicators that can be used to optimize anesthesia management. Moderate or deep sedation in operating room anesthesia and intensive care units driven by EEG monitors may become a standard practice. Therefore, electroencephalography can be used to optimize anesthesia management, which may become a standard practice for future anesthesia and sedation management [3]. Saito used a repeated measure design to study the ECG responses under 1% halothane anesthesia, 1.5% isoflurane nitrous oxide anesthesia, and awake state by comparing the ECG responses and exploring the effects of different anesthetics on ECG indicators, providing good research information on anesthesia techniques [4]. The above research involves multiple research fields of anesthesia technology and provides guidance for the research of this topic. These literature did not conduct joint research on anesthesia technology and neuroscience, which limited the in-depth research of this topic.

After further literature review, the following research literature on anesthesia technology and neuroscience was found. Mangia pointed out that more and more otorhinolaryngological operations have adopted sedative local anesthesia, which may be related to reducing the incidence rate and cost of surgery. He discussed the feasibility of facial nerve monitoring in otologic surgery. Although facial nerve monitoring is usually used for surgery under general anesthesia, objective reports show that intraoperative facial nerve monitoring under local anesthesia and sedation is feasible and reliable. As the level of anesthesia increases, a gradually decreasing low amplitude baseline value is usually obtained, and isolated oscillations can be distinguished from those of surgical procedures or nerve electrical stimulation. Therefore, intraoperative facial nerve monitoring is feasible under less active anesthesia management, and the need for intraoperative nerve monitoring should not become an obstacle to otology surgery [5]. Nunes emphasized the importance of wise selection of anesthetics and evaluated the effects of anesthesia depth, hemodynamic status, and other factors that may interfere with signal capture during intraoperative neurophysiological monitoring. In addition, he pointed out that due to the advancement of neural monitoring technology in recent years, it is possible to gain a deeper understanding of neural function during anesthesia during surgery and evaluate intraoperative awareness and neural integrity in real-time. Neurophysiological monitoring can be used for surgeries that pose risks to targeted nerve tissue and adjacent structures, which can be highly sensitive and specific in linking surgical procedures with neurophysiological changes, and identify risk situations before surgery to prevent postoperative neurological deficits [6]. Through reading, it is evident that scholars have provided valuable insights into the research direction. However, their research mainly focuses on theoretical research and lacks empirical verification. In order to optimize this issue, the aim is to conduct more data research and analysis to strengthen their research.

In this article, anesthesia status data was monitored and analyzed using a neuroscience-based anesthesia status monitoring algorithm. The experiment showed that the average value of information entropy of EEG signal in conscious state, mild anesthesia state, and severe anesthesia state was 0.794, 0.573, and 0.3975, respectively. With the increase of the depth of anesthesia, the information entropy of EEG signals gradually decreased, the complexity decreased, and the intensity reflecting the brain neural activity decreased. In addition, the average oxygen saturation in conscious state, mild anesthesia state, and severe anesthesia state was 97.97, 93.50, and 90.71%, respectively, which indicated that with the deepening of anesthesia, the patients’ oxygen saturation would decrease. The innovation of this article lies in the use of neuroscience-based monitoring and analysis of anesthesia depth status data. Compared to traditional anesthesia status monitoring methods, this method can provide a more comprehensive understanding of the patient’s brain neural activity and blood oxygen levels at different anesthesia depths, and achieve more accurate monitoring of anesthesia status.

## 2 Evaluation on monitoring anesthesia depth status data

### 2.1 Monitoring aspects of anesthesia status data

Anesthesia status data monitoring plays an important role in modern anesthesia medicine, as it can improve surgical safety and patient’s quality of life [7,8]. This monitoring refers to the monitoring and recording of the patient’s physiological parameters during the anesthesia process to ensure that the patient is in a safe anesthesia state during surgery. By monitoring the physiological parameters of patients, anesthesiologists can promptly detect and handle abnormal situations that occur during anesthesia, in order to ensure the safety of patients. Anesthesia status data monitoring usually requires monitoring the following aspects.

#### 2.1.1 Consciousness level

Consciousness level refers to a person’s level of awakening and cognitive state, which can be evaluated through various methods, such as observing a patient’s facial expressions, language responses, motor responses, etc. [9,10]. During the anesthesia process, monitoring the level of consciousness is very important, as changes in the depth of anesthesia directly affect the safety and success rate of the surgical process. The following is a detailed explanation of the different levels of consciousness.

Awakening state refers to the patient’s complete awakening and clear state of consciousness, which allows them to make appropriate responses and reactions. Before and after anesthesia surgery, a clear state needs to be maintained.

Drowsiness refers to the patient being in a very mild state of anesthesia, but still able to breathe autonomously and have certain reactions. The drowsiness state is usually used during the sedation stage before surgery to make the patient feel comfortable and relaxed.

Sedative state refers to a conscious and relatively deep state of anesthesia in which the patient’s conscious state weakens, but can still be awakened. It is usually used during surgery to make patients feel comfortable and relaxed, while also reducing pain and discomfort during the surgery process.

Unconscious state refers to the state in which a patient is completely unconscious and unable to respond in any way, requiring the use of auxiliary equipment such as a ventilator for breathing and maintaining life functions. It needs to be used during surgery to ensure safe operation in the surgical department.

#### 2.1.2 Breathing

Breathing is one of the important functions of the human body to maintain life, and monitoring respiratory changes is also very important in monitoring anesthesia depth status data. The following is a detailed explanation of the different aspects of breathing.

Breathing depth: The depth of exhalation and inhalation can usually be determined by the breathing amplitude. During anesthesia, changes in respiratory depth may affect the exchange of oxygen and carbon dioxide in the lungs, leading to respiratory dysfunction.

Respiratory rate refers to the number of breaths per unit time. The respiratory rate of normal adults is 12–20 times per minute. During anesthesia, the change of respiratory rate usually reflects the influence of anesthetic drugs on respiratory control.

Respiratory rhythm refers to the regularity of breathing, including the pause time between exhalation, inhalation, and breathing. Abnormal respiratory rhythm may lead to problems such as alveolar collapse and hypoxia.

#### 2.1.3 Circulation system

The circulatory system is a human system composed of the heart, blood vessels, and blood. Its main function is to transport oxygen and nutrients to various parts of the body, while also transporting metabolic products and carbon dioxide from within the body to the lungs and kidneys for excretion. By monitoring the status of the circulatory system, the degree of anesthesia can be analyzed. The following is a detailed explanation of the different aspects of the loop.

Heart rate refers to the number of beats per minute of the heart, with a normal adult heart rate of 60–100 beats per minute. During anesthesia, changes in heart rate may reflect the impact of anesthetic drugs on the heart, and the occurrence of heart rate abnormalities may lead to problems such as poor blood flow and impaired cardiac function.

Blood pressure refers to the pressure of blood flow on the walls of blood vessels, including two values: systolic and diastolic blood pressure. During anesthesia, changes in blood pressure may be influenced by anesthetic drugs and surgical stimuli.

Oxygen saturation refers to the amount of oxygen in the blood, usually expressed as oxygen saturation. Under normal circumstances, the oxygen saturation should be above 95%, and below 90% may lead to hypoxia.

#### 2.1.4 Muscle state

During the surgery process, muscle relaxants can help doctors perform the surgery better and avoid affecting the quality and safety of the surgery due to the patient’s muscle resistance. The following is a detailed explanation of muscle status.

Degree of muscle relaxation: During anesthesia, the use of muscle relaxants can cause muscle relaxation. Improper use may lead to muscle stiffness or inability to move during consciousness recovery.

Muscle resistance: After the action of anesthetic drugs, some patients may experience muscle resistance, which means that muscle contraction is difficult to be completely suppressed. At this point, the doctor needs to reassess the degree of muscle relaxation and adjust the medication use and dosage.

#### 2.1.5 Pupillary response

The pupils are black circles in the eyes, through which light can enter. Pupil response refers to the automatic adjustment of the pupils to changes in light. Under normal circumstances, when light shines on the eyes, the pupils would quickly contract to limit excessive light from entering the eye and causing damage. Under deep anesthesia, due to the suppression of nervous system function, the contraction response of the pupils would weaken or disappear. Therefore, monitoring the pupillary response can help to understand the patient’s level and depth of anesthesia, adjust the dosage of anesthesia in a timely manner, and handle sudden situations to ensure anesthesia safety. The following is a detailed explanation of the pupil response.

Pupil size: Under normal circumstances, a person’s pupil size ranges from 2 to 6 mm. The size of the pupils is influenced by various factors, such as light intensity and surrounding environment.

Response to light: This refers to the reaction of the pupils to light. Normally, when light strikes an eye, the pupil of that eye contracts to protect the retina from damage. The pupils of the other eye would slightly dilate to adapt to dark environments.

Pupil reflex: Pupil reflex refers to the automatic contraction and dilation of the pupils caused by light shining on the eyes. By observing changes in pupil size, doctors can determine whether a patient is under anesthesia and whether the nervous system is functioning properly.

**Figure 1:**
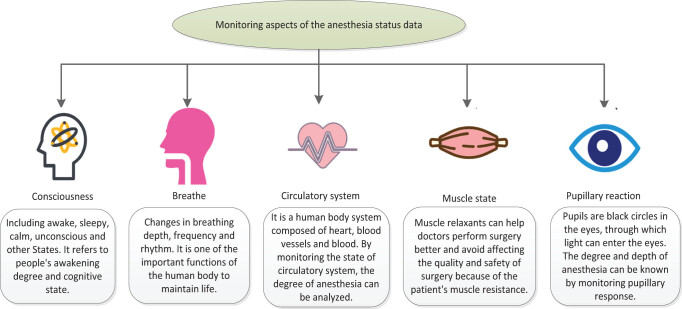
Monitoring aspect of anesthesia status data.

The above aspects can be monitored using professional instruments to help doctors determine whether the depth of anesthesia has reached an appropriate level. Monitoring anesthesia depth status data can effectively improve the safety and success rate of anesthesia surgery. The monitoring aspect of anesthesia status data is shown in Figure 1.

### 2.2 Monitoring process of anesthesia status data

In anesthesia operations, monitoring anesthesia status data is very important. It can help anesthesiologists detect changes in the patient’s vital signs and anesthesia depth in a timely manner, and take necessary measures to ensure the patient’s safety [11,12]. Therefore, the monitoring process of anesthesia status data needs to strictly follow the prescribed processes and standards to ensure the accuracy and reliability of monitoring. Anesthesiologists should be proficient in the techniques and methods of monitoring anesthesia status, timely record monitoring data, analyze and judge monitoring data, take corresponding anesthesia control measures, and ensure that patients are in a safe state during the anesthesia process. The monitoring process of anesthesia status data mainly includes the following steps.

#### 2.2.1 Pre-anesthesia assessment

Pre-anesthesia assessment refers to a detailed assessment of a patient’s physical condition by medical personnel before undergoing anesthesia, in order to determine whether anesthesia is suitable and which anesthesia method to choose. At the same time, it can also identify some risk factors related to anesthesia [13,14]. Pre-anesthesia assessments are usually completed by anesthesiologists or other professional healthcare personnel.

During the pre-anesthesia assessment, medical staff need to collect the patient’s medical history and family history information, including the patient’s past medical history, family medical history, surgical experience, allergies, smoking, alcohol consumption, drug use, and so on, and whether the patient has special needs, such as arranging the ventilator in advance. Medical staff also need to understand the patient’s current physical condition, including vital signs, height, weight, respiratory status, heart condition, liver and kidney function, blood test results, etc.

Based on the results of the pre-anesthesia assessment, medical staff would assess the patient’s anesthesia risk and develop corresponding plans [15]. If a patient is found to have a high risk of anesthesia, necessary measures need to be taken to handle it, such as selecting safer anesthesia methods, adjusting the amount of anesthesia, and regularly monitoring vital signs.

#### 2.2.2 Anesthesia induction

Anesthesia induction refers to the process of injecting a certain dose of anesthetic into a patient before undergoing medical procedures such as surgery, causing them to lose consciousness, become insensitive to external stimuli, relax their muscles, and feel painless. This process requires a detailed evaluation by professional medical personnel based on the patient’s physical condition and the type of surgery.

During the anesthesia induction process, medical staff would inject drugs into the patient to put them into a state of anesthesia. The choice of medication would depend on the patient’s physical condition, surgical type, and the physician’s personal experience. Usually, anesthetic drugs can be divided into two types: intravenous anesthesia and inhalation anesthesia.

In addition, medical personnel need to closely monitor the patient’s awareness level, breathing, heart rate, and other indicators to ensure that the patient is in a safe state to complete the surgery. If the dosage of anesthetic drugs is insufficient, patients may wake up during the surgery and feel pain and discomfort. If the dosage is too large, it may lead to respiratory depression and other complications, and even endanger the patient’s life.

#### 2.2.3 Anesthesia maintenance

Anesthesia maintenance refers to the continuous monitoring of the patient’s vital signs and depth of anesthesia after the patient enters the anesthesia state. Medical personnel need to adjust the dosage and method of anesthesia medication to maintain the patient in an appropriate anesthesia state. At the same time, medical personnel should also pay attention to monitoring the patient’s respiratory and circulatory functions to avoid intraoperative accidents and complications. In addition, medical personnel also need to pay attention to the comfort of patients, adjust the operating room environment and posture, and other factors to minimize patient’s discomfort and pain, and improve surgical effectiveness and safety. In summary, anesthesia maintenance is a crucial task that directly affects the patient’s life safety and surgical quality.

#### 2.2.4 Anesthesia relief

After the surgery is completed, medical staff need to take a series of measures to help patients recover smoothly from anesthesia, ensuring that they can smoothly return to a conscious state and have stable physical indicators, and facilitating the smooth return to the ward for further treatment. These measures include observing the patient’s vital signs, monitoring the depth of anesthesia, and alleviating pain, which helps medical personnel identify and handle potential risks in a timely manner, and improves the safety and success rate of the anesthesia relief process.

#### 2.2.5 Post-anesthesia observation

Post-anesthesia observation refers to the need for medical personnel to conduct detailed observation and evaluation of patients after receiving anesthesia treatment. This process is very important as it can help identify and promptly address potential complications or sequelae. During anesthesia, the vital signs of patients may be affected, including changes in breathing, circulation, nervous system, and other aspects. If medical personnel do not observe and evaluate in a timely manner, corresponding measures cannot be taken in a timely manner, which may worsen the patient’s health condition and even endanger their life. Therefore, post-anesthesia observation is an indispensable part of the anesthesia treatment process. Meanwhile, during the post-anesthesia observation process, medical personnel also need to evaluate the metabolism and excretion of anesthetic drugs to determine when the patient can be safely discharged.

## 3 Evaluation on monitoring anesthesia status

### 3.1 Application of neuroscience in anesthesia technology

Neuroscience is the discipline that studies the structure, function, and development of the nervous system. It involves multiple levels, from molecular and cellular level research, physiological and behavioral experiments, to imaging research of the nervous system, and the development of computer models. Research in this field has helped researchers better understand the mechanisms of the brain and other nervous systems, thereby driving the development of many fields related to nerves, such as cognitive neuroscience, neuropharmaceutics, neurological diseases, and so on. Neuroscience plays an important role in exploring the fundamental research of the nervous system, and also has a significant impact on application fields such as neuromedicine and neuroengineering. The application of neuroscience in anesthesia technology mainly involves two aspects: one is to understand the neural mechanisms of anesthesia, and the other is to evaluate and regulate the depth of anesthesia through neural monitoring.

First, neuroscience has helped researchers better understand the neural mechanisms of anesthesia. Anesthetic drugs can affect signal transmission between neurons, thereby reducing consciousness levels and sensitivity to external stimuli. Research in neuroscience has revealed the molecular basis and neural circuit mechanisms of these effects, providing guidance for the search for more effective and safer anesthetic drugs.

Second, neural monitoring technology can effectively evaluate and regulate the depth of anesthesia. Neuromonitoring can monitor the depth of anesthesia through neuroelectrophysiological techniques (such as electroencephalography, spinal cord evoked potential, etc.) or physiological monitoring (such as respiration, blood pressure, heart rate, etc.), and adjust medication based on the monitoring results to achieve more accurate and safe anesthesia effects. In addition, neural monitoring can help anesthesiologists better understand the patient’s neurological condition and avoid potential risks of neurological damage.

The application of neuroscience in anesthesia technology provides a deeper and more comprehensive understanding and control of anesthesia effects for medical surgeries, which helps to improve the safety and effectiveness of anesthesia.

### 3.2 Anesthesia status monitoring algorithm based on neuroscience

In pharmacodynamic models, the S-type maximum effect chamber model can be used to evaluate the monitoring of anesthesia status data. The formula is

(1) \[E=\frac{{E}_{\max }\times {C}^{{\mathrm{s}}}}{{\mathrm{E}}{{\mathrm{C}}}_{50}^{{\mathrm{s}}}+{C}^{{\mathrm{s}}}}+{E}_{0},]\]

where *s* is the steepness constant. When studying the effects of anesthetic drugs, an S-type maximum effect chamber model with nonlinear inhibition is used, and is represented as follows:

(2) \[E={E}_{\max }-({E}_{\max }-{E}_{\min })\times \frac{{C}_{{\mathrm{e}}}^{{\mathrm{s}}}}{{\mathrm{E}}{{\mathrm{C}}}_{50}^{{\mathrm{s}}}+{C}_{{\mathrm{e}}}^{{\mathrm{s}}}},]\]

where \[{E}_{\max }]\] is the maximum drug effect size, \[{E}_{\min }]\] is the minimum drug effect size, \[{\mathrm{E}}{{\mathrm{C}}}_{50}^{{\mathrm{s}}}]\] is the value corresponding to 50% of the maximum effective concentration of anesthetic drugs, and \[{C}_{{\mathrm{e}}}^{{\mathrm{s}}}]\] is the drug concentration value of the effect chamber.

Then, it is necessary to quantitatively describe the drug characteristics. Under the condition of constant blood drug concentration, the time required when the drug concentration in the effect chamber reaches half of the blood drug concentration is called the half-life, and the formula is

(3) \[{T}_{1/2}=\mathrm{ln}2/{k}_{{\mathrm{e}}}.\hspace{1em}]\]

Here \[{k}_{{\mathrm{e}}}]\] is the first-order rate constant for the elimination of the drug effect chamber.

The relationship between blood concentration and effect chamber concentration can be described as

(4) \[{\mathrm{d}}{C}_{{\mathrm{e}}}/{\mathrm{d}}t={k}_{{\mathrm{e}}}{[}{C}_{{\mathrm{t}}}-{C}_{{\mathrm{e}}}],]\]

where \[{C}_{{\mathrm{t}}}]\] is the concentration of end-of-life drugs in the blood. The model estimates the concentration of anesthetic effect chamber \[{C}_{{\mathrm{e}}}]\] by taking a series of different values for \[{k}_{{\mathrm{e}}}]\] . When the initial value of \[{k}_{{\mathrm{e}}}]\] is determined, the best \[{k}_{{\mathrm{e}}}]\] can be evaluated by the maximum correlation coefficient of \[{R}^{2}]\] . The principle of evaluation is to recognize that the measured effect size and the modeled effect size obey the maximum correlation coefficient:

(5) \[{R}^{2}=1-\frac{{\mathrm{SE}}}{{\mathrm{ST}}}=1-\frac{\mathop{\sum }\limits_{i=1}^{n}{({y}_{i}-{\hat{y}}_{i})}^{2}}{\mathop{\sum }\limits_{i=1}^{n}{({y}_{i}-{\bar{y}}_{i})}^{2}}.]\]

In the above formula, \[{\mathrm{SE}}]\] is the sum of squares of the difference between the actual quantity \[{y}_{i}]\] and the model predicted quantity \[{\hat{y}}_{i}]\] at a given time, and \[{\mathrm{ST}}]\] is the sum of squares of the difference between the actual value and all actual averages \[{\bar{y}}_{i}]\] .

This algorithm can accurately evaluate the depth and state of anesthesia of patients by monitoring and analyzing these signals, helping doctors better control the amount of anesthesia used, thereby reducing the occurrence of adverse reactions. In addition, the algorithm can also provide a scientific basis for clinicians to guide clinical anesthesia operations, so as to better protect the safety of patient safety.

## 4 Experimental monitoring of anesthesia depth status data based on neuroscience

In this section, in order to evaluate the effectiveness and reliability of anesthesia state monitoring algorithms based on neuroscience in practical applications, performance testing is needed to deeply understand the specific performance of the algorithm in anesthesia depth state monitoring, thereby providing strong support for its further optimization and improvement.

**Figure 2:**
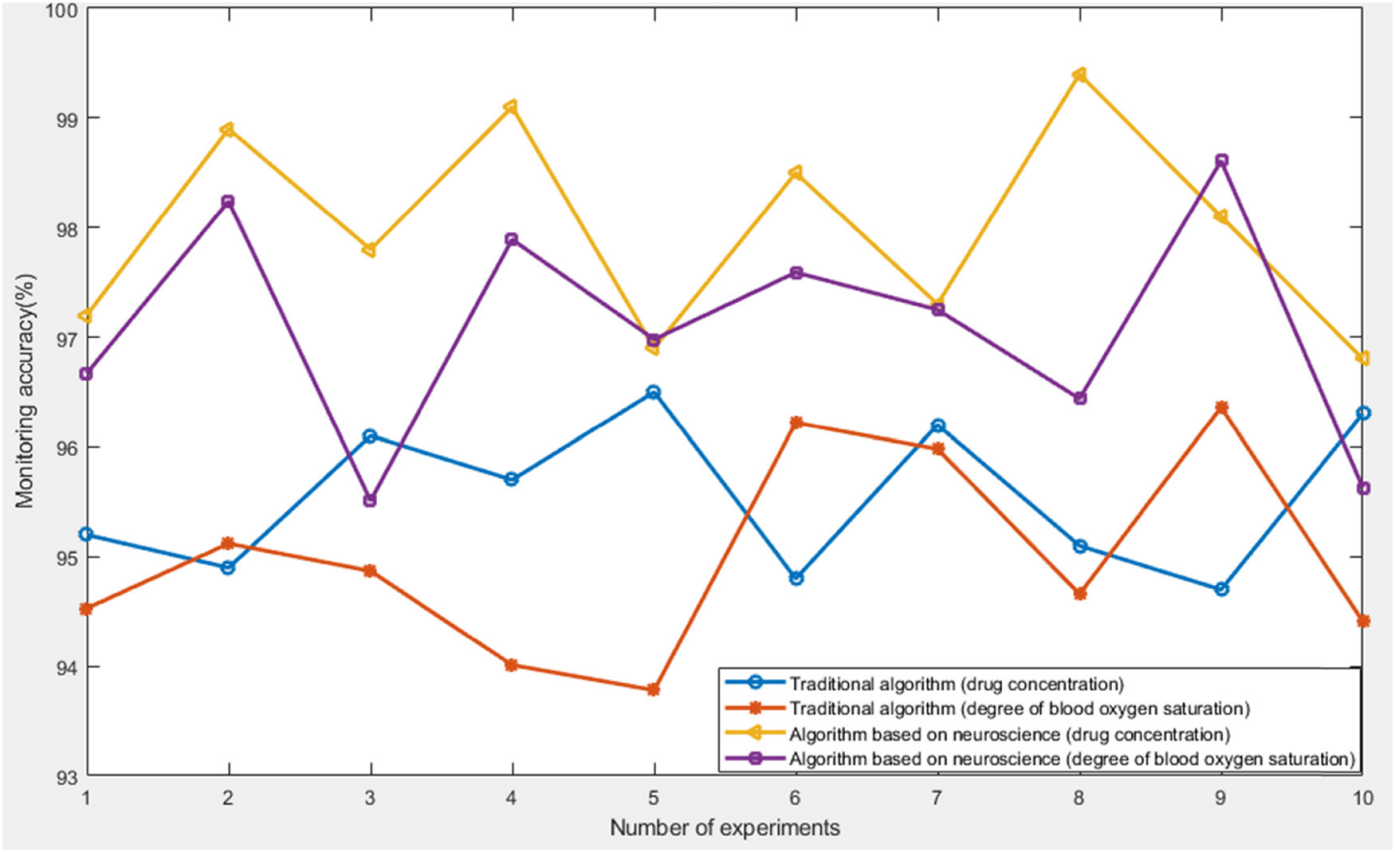
Monitoring accuracy of two algorithms.

The first is to test the accuracy of monitoring drug concentration and oxygen saturation in patients’ blood based on neuroscience anesthesia state monitoring algorithm and traditional anesthesia state monitoring algorithm. The detailed data is shown in Figure 2.

From the data in Figure 2, it can be seen that compared to traditional anesthesia status monitoring algorithms, neuroscience-based anesthesia status monitoring algorithms had higher monitoring accuracy. After calculation, the average monitoring accuracy of the traditional anesthesia state monitoring algorithm for drug concentration and oxygen saturation was 95.55 and 95.00%, respectively. The average monitoring accuracy of anesthesia state monitoring algorithm based on neuroscience for drug concentration and oxygen saturation was 98.00 and 97.09%, respectively. This experiment proved that the monitoring performance of anesthesia status monitoring algorithms based on neuroscience was more excellent.

**Figure 3:**
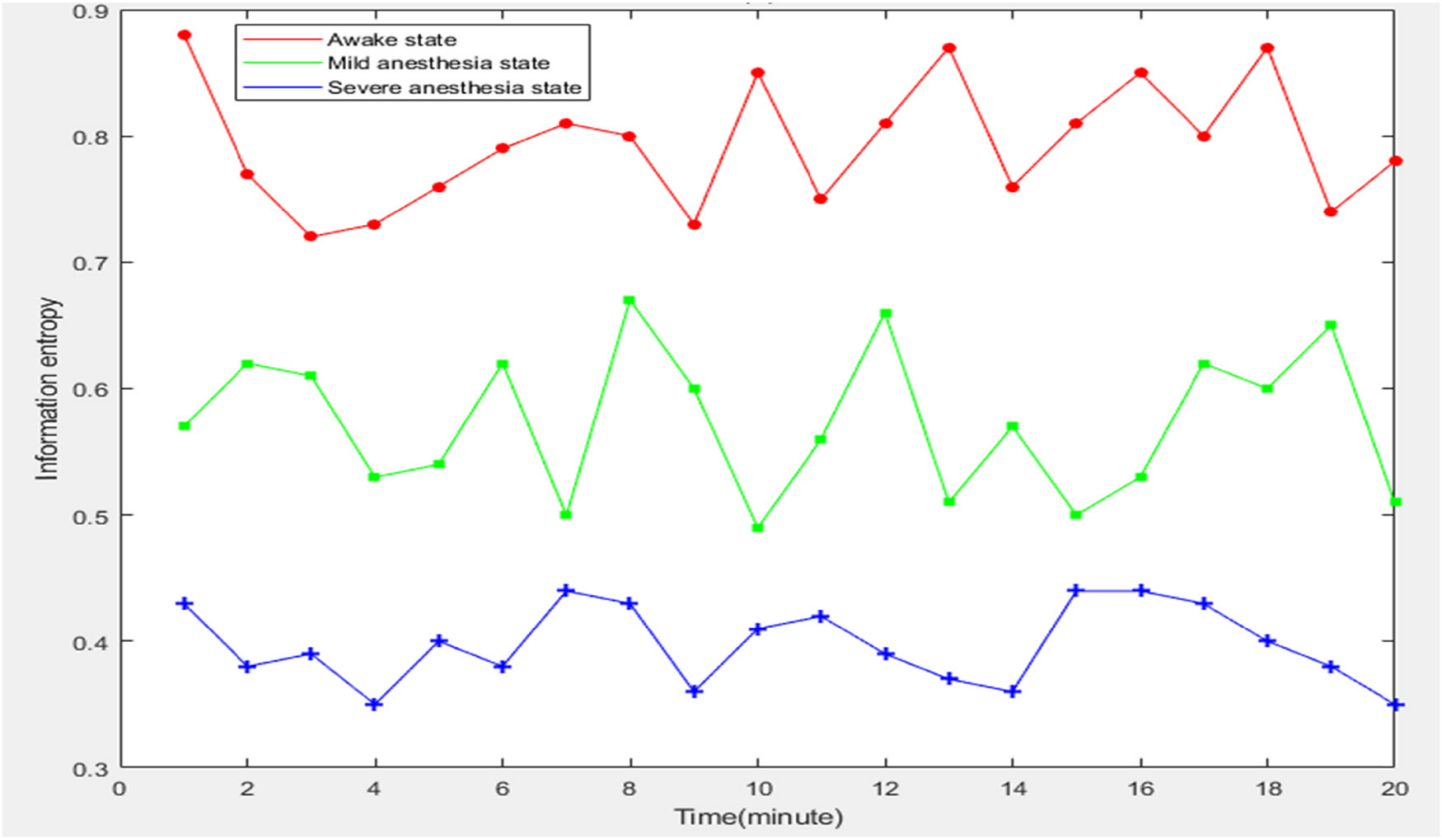
Information entropy of EEG signals in patients with different anesthesia states.

During the anesthesia process, as the degree of anesthesia deepens, the EEG signals change from active disorder to regular and stable. Therefore, applying the information entropy theory to the analysis of anesthesia EEG signals can accurately reflect the depth of anesthesia. In short, the more complex the EEG signal, the greater the information entropy. The more stable the EEG signal is, the smaller the information entropy value is. Therefore, in the following experiment, the information entropy of EEG signals of patients with different anesthesia states would be tested and counted to explore the change trend of anesthesia depth state and brain nerve signals. The detailed data is shown in Figure 3.

From the data in Figure 3, it was obvious that there was a large difference in the information entropy of EEG signals among patients in awake state, mild anesthesia state, and severe anesthesia state. After calculation, the average information entropy of EEG signals of patients in awake state, mild anesthesia state, and severe anesthesia state was 0.794, 0.573, and 0.3975, respectively. This showed that with the increase of the depth of anesthesia, the information entropy value of EEG signals decreased, the complexity of EEG signals decreased, and the brain neural activity decreased.

**Figure 4:**
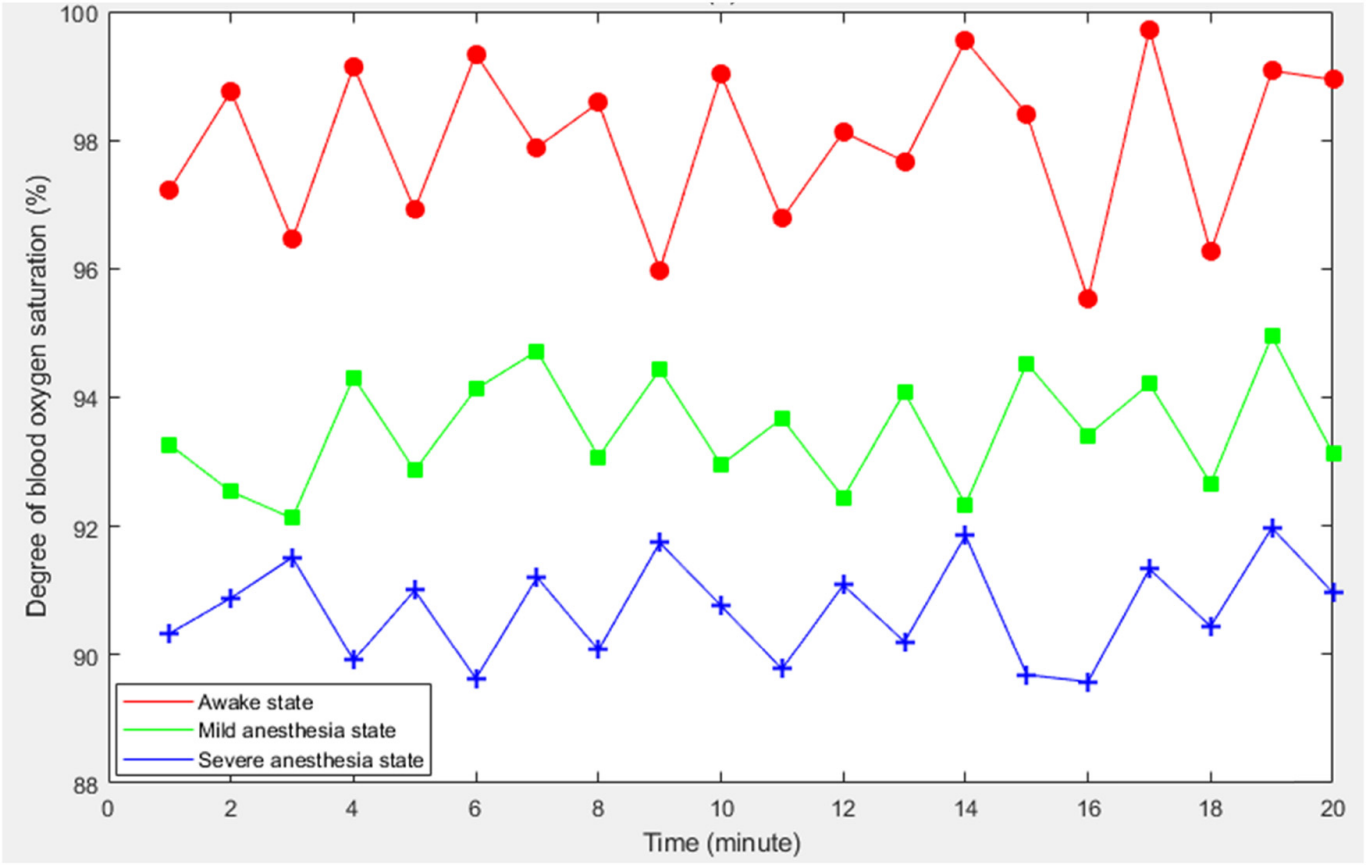
Oxygen saturation of patients in different anesthesia states.

Similarly, oxygen saturation is also an important analytical indicator of anesthesia depth data monitoring. Therefore, in the following experiments, oxygen saturation of patients in different anesthesia states would be tested and counted to explore the change trend of anesthesia depth state and oxygen saturation. The detailed data is shown in Figure 4.

From the data in Figure 4, it can be seen that the oxygen saturation of patients under different anesthesia conditions had a relatively large change. After calculation, the average oxygen saturation of patients in awake state, mild anesthesia state, and severe anesthesia state was 97.97, 93.50, and 90.71%, respectively. This indicated that with the deepening of anesthesia depth, the patient’s oxygen saturation would decrease.

This is because in the state of deep anesthesia, the metabolic rate of the body slows down, vital capacity decreases, respiration becomes shallow and slow, leading to insufficient oxygen supply, which leads to the reduction of oxygen saturation. Therefore, it is crucial to monitor and maintain the patient’s blood oxygen levels during anesthesia to avoid serious complications.

## 5 Conclusions

The monitoring and analysis of anesthesia depth status data based on neuroscience refers to the precise monitoring and analysis of anesthesia depth status by studying the impact of anesthesia depth status on brain neural activity. Compared with traditional anesthesia monitoring methods, neuroscience based monitoring and analysis of anesthesia depth status data has higher accuracy and reliability, providing doctors with more accurate monitoring and analysis results of anesthesia depth status to ensure the safety and success of the surgical process. Through the neuroscience-based anesthesia status monitoring algorithm, the anesthesia depth status is more accurately monitored, and the EEG information entropy and oxygen saturation of patients with different anesthesia status can be obtained, ensuring the safety of anesthesia surgery. Experiments have proved that during anesthesia, EEG signals would change with the amount of anesthetic. The deeper the depth, the lower the complexity and information entropy of EEG signals. The physiological information contained in EEG signals under different anesthesia states also varies. EEG signals are very sensitive to anesthetics, and as anesthesia deepens, the EEG signals become more and more stable. In addition, in the state of deep anesthesia, the metabolic rate of the body slows down, vital capacity decreases, and oxygen saturation decreases. Therefore, it is crucial to monitor and maintain the patient’s blood oxygen levels during anesthesia to avoid serious complications. In summary, the monitoring and analysis of anesthesia depth status data based on neuroscience is a beneficial method for anesthesia depth monitoring, which would be more widely applied and promoted in the future. However, there are still some shortcomings that need to be further addressed in the monitoring and analysis of anesthesia depth status data based on neuroscience. For example, current monitoring methods mainly rely on physiological indicators such as EEG signals, but these indicators are influenced by multiple factors, making it difficult to accurately monitor each patient. In addition, current monitoring equipment is often cumbersome and difficult to use in real-time in scenarios such as operating rooms, resulting in limited monitoring effectiveness. Therefore, future research needs to address these issues, and improve the accuracy and real-time nature of monitoring, in order to better ensure the safety and health of patients.
